# Comparative Diagnostic Yield of Cytology, Imprint Cytology, and Histopathology in Medical Thoracoscopic Pleural Biopsies: A Prospective Observational Study

**DOI:** 10.7759/cureus.92211

**Published:** 2025-09-13

**Authors:** Varuna Jethani, Amita Mason, Sushant Khanduri, Rakhee Khanduri, Aarti Kotwal, Sumit Garg, Sumit Jethani

**Affiliations:** 1 Respiratory Medicine, Himalayan Institute of Medical Sciences, Swami Rama Himalayan University, Dehradun, IND; 2 Microbiology, Himalayan Institute of Medical Sciences, Swami Rama Himalayan University, Dehradun, IND; 3 Pathology, Himalayan Institute of Medical Sciences, Swami Rama Himalayan University, Dehradun, IND; 4 Community Medicine, North Delhi Municipal Corporation (DMC) Medical College and Hindu Rao Hospital, Delhi, IND

**Keywords:** histopathology, imprint cytology, pleural biopsy, pleural effusion, thoracoscopy

## Abstract

Background

Pleural effusion is a common clinical problem with varied etiologies. Timely diagnosis is essential for appropriate treatment. This study aimed to compare the diagnostic performance of brush cytology and imprint cytology, with histopathology, in pleural biopsy specimens obtained via thoracoscopy.

Methods

This prospective observational study included 96 patients with undiagnosed exudative pleural effusion undergoing medical thoracoscopy. Biopsy samples were analyzed by histopathology, brush cytology, and imprint cytology. Diagnostic metrics, including sensitivity, specificity, positive predictive value (PPV), negative predictive value (NPV), McNemar’s test, receiver operating characteristic (ROC) analysis, and logistic regression, were computed.

Results

Imprint cytology demonstrated higher sensitivity (73.17%) and NPV (76.60%) compared to brush cytology (65.85% and 72.00%, respectively). Logistic regression showed that a positive imprint result predicted biopsy positivity with an odds ratio of 5.17. ROC analysis confirmed superior diagnostic performance for imprint cytology.

Conclusion

Imprint cytology is a reliable, rapid, and effective diagnostic tool in thoracoscopic pleural biopsy evaluation, with performance metrics approaching those of histopathology.

## Introduction

Pleural effusion is a common clinical problem encountered in respiratory medicine. It is caused by over 60 conditions, ranging from benign to malignant etiologies. Accurate and timely diagnosis is crucial to guide appropriate management. While thoracentesis and pleural fluid analysis are often the initial diagnostic steps, they have limited sensitivity in identifying the underlying etiology - especially in malignant or tuberculous effusions.

Medical thoracoscopy (MT) has become a vital diagnostic tool for undiagnosed pleural effusions. In high tuberculosis burden countries like India, patients are often empirically initiated on antitubercular therapy based on pleural fluid analysis alone; however, this approach may be misleading, as some cases initially presumed to be tuberculous pleural effusion are later diagnosed as malignant following MT. Therefore, it is crucial to confirm the diagnosis of tuberculosis through pleural biopsy obtained via MT, to ensure appropriate and accurate treatment. MT provides direct visualization of the pleura and permits targeted biopsies under vision, thereby improving diagnostic yield compared to blind or image-guided techniques; moreover, imprint cytology performed during the thoracoscopic procedure offers a rapid, reliable adjunct that further enhances diagnostic accuracy [1]. While histopathology remains the gold standard for evaluating pleural biopsy specimens, the inherent delay in processing and reporting may postpone timely management. In this context, cytological techniques such as imprint cytology have been shown to be rapid, accurate, and reliable alternatives for early diagnosis [2]. Imprint cytology involves pressing the biopsy specimen onto a glass slide, staining, and rapid microscopic examination. It has been shown in some studies to provide high sensitivity and specificity in detecting malignancy and granulomatous inflammation, with quicker turnaround time than histopathology [3,4].

Several studies have explored the comparative diagnostic utility of these modalities. Hantera et al. demonstrated that imprint cytology is highly concordant with histopathological diagnosis in malignant pleural diseases [1], while Sharma et al. emphasized the use of cytological evaluation as a valuable adjunct [2]. In resource-limited settings, where pathology infrastructure may be constrained, a diagnostic method that is both efficient and cost-effective is essential. Thus, evaluating and comparing the diagnostic yields of cytology, imprint cytology, and histopathology in pleural biopsies obtained via thoracoscopy becomes clinically relevant. Additionally, an analysis by Heine et al. supports the complementary roles of cytology and histology in pleural biopsy [5].

Objectives

The main objectives of this study are: (I) to compare the diagnostic yield of cytology, imprint cytology, and histopathology in pleural biopsy specimens obtained during MT; (II) to determine the sensitivity, specificity, and diagnostic accuracy of imprint cytology compared to histopathology; and (III) to evaluate the clinical utility and turnaround time of imprint cytology in facilitating early diagnostic decision-making

## Materials and methods

Study design and period

This was a prospective, cross-sectional, observational study conducted at the Department of Respiratory Medicine, Himalayan Institute of Medical Sciences, Dehradun, India, from October 2024 to April 2025.

Inclusion criteria

Adult patients aged above 20 years were included to ensure a homogeneous adult cohort. Patients presenting with exudative pleural effusion based on Light's criteria of undiagnosed etiology were enrolled. Only patients suitable for and consenting to undergo rigid MT were included.

Exclusion criteria

Patients with cardiovascular instability or significant comorbidities were excluded. Patients with severe respiratory compromise (PaO₂ < 60 mmHg or PaCO₂ > 50 mmHg) were excluded. Known cases of recent myocardial infarction (within the past three months) were excluded. Patients with severe COPD (FEV₁ < 1 L or <35% predicted) were excluded. Patients with coagulopathy or contraindications to thoracoscopy were excluded.

Data collection

Following informed consent, each patient underwent diagnostic rigid thoracoscopy under local anesthesia and conscious sedation. Multiple pleural biopsy samples (three to four, in formalin for histopathology) were obtained. Additionally, brush cytology and imprint cytology smears were prepared intraoperatively. Imprint cytology was performed by gently pressing the biopsy tissue on clean glass slides, which were then fixed and stained using rapid hematoxylin-eosin methods for early cytological interpretation.

Statistical analysis

All statistical analyses were performed using IBM SPSS Statistics for Windows, Version 25 (Released 2017; IBM Corp., Armonk, NY, USA). Diagnostic accuracy was evaluated using sensitivity, specificity, positive predictive value (PPV), negative predictive value (NPV), and receiver operating characteristic (ROC) curve analysis. McNemar’s test was used to compare paired proportions. Binary logistic regression was applied to estimate the odds ratio and model fit indicators, including Pseudo R², Akaike information criterion (AIC), and log-likelihood. A p-value of <0.05 was considered statistically significant.

Ethical considerations

The study was conducted in accordance with the Declaration of Helsinki. Ethical approval was obtained from the Institutional Ethics Committee of Himalayan Institute of Medical Sciences (Approval ID: HIMS/RC/2025/07). Written informed consent was obtained from all patients prior to enrollment.

## Results

A total of 96 patients with undiagnosed exudative pleural effusion were included in the study. The mean age of participants was 54.4 years, ranging from 13 to 87 years. Among them, 63 were male (65.6%), and 33 were female (34.4%). All patients underwent diagnostic MT, and pleural biopsy specimens were subjected to histopathological examination, which served as the reference standard for diagnosis. Simultaneously, the specimens were evaluated using imprint cytology and brush cytology to assess their diagnostic yield and correlation with biopsy-confirmed findings.

**Figure 1:**
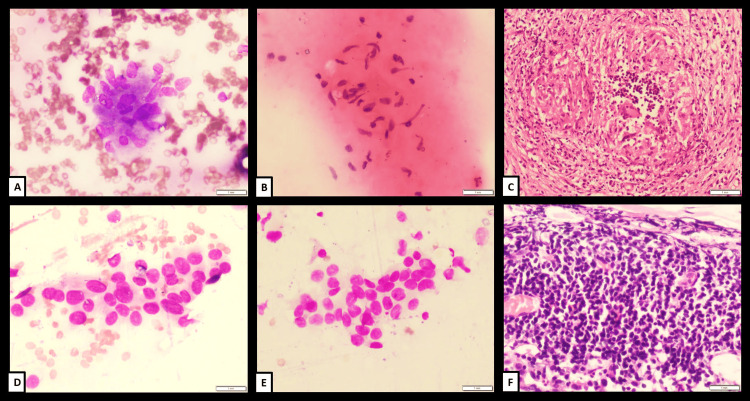
(A-C) Case 1 and (D-F) Case 2 Case 1 (40/M patient; A to C): Brush cytology smear (A; MGG, 400X) and imprint cytology smear (B; H&E, 400X) show epithelioid histiocytic clusters suggestive of granulomatous pathology. Histopathological examination (C; H&E, 200X) revealed necrotizing granulomatous pathology. Case 2 (72/F patient; D to F): Brush cytology smear (D; H&E, 400X) and imprint cytology smear (E; H&E, 400X) show atypical cell clusters with round cell morphology, suggestive of small cell carcinoma. Histopathology examination (F; H&E, 200X) revealed a malignant small round cell tumour with crush artefact, suggestive of small cell carcinoma. MGG, May-Grünwald-Giemsa; H&E, Hematoxylin and Eosin

The diagnostic performance of each method was first evaluated in terms of sensitivity, specificity, PPV, and NPV. Imprint cytology demonstrated a sensitivity of 73.17% (N = 41), higher than brush cytology, which showed a sensitivity of 65.85% (N = 41) (Table 1). Both modalities shared a similar specificity of 65.45% (N = 55). The PPV of imprint cytology was 61.22% (N = 49), while that of brush cytology was slightly lower at 58.70% (N = 46). The NPV of imprint cytology was also marginally better (76.60%, N = 47), compared to brush cytology (72.00%, N = 50). These metrics suggest that imprint cytology is more reliable in ruling out pleural pathology and has a higher probability of correctly identifying disease when present (Figure 1).

**Table 1:** Diagnostic Performance Metrics of Cytological Techniques

Cytology Type	Sensitivity (N, %)	Specificity (N, %)	PPV (N, %)	NPV (N, %)
Brush Cytology	41/62 (65.85%)	55/84 (65.45%)	46/78 (58.70%)	50/69 (72.00%)
Imprint Cytology	41/56 (73.17%)	55/84 (65.45%)	49/80 (61.22%)	47/61 (76.60%)

To assess agreement with histopathological diagnosis, confusion matrices were constructed for both cytological techniques. For brush cytology, 27 patients who had biopsy-confirmed disease were also brush-positive, while 14 biopsy-positive cases were missed (false negatives).

Based on the confusion matrices against biopsy: (1) Brush cytology - False negatives (14 cases): These were biopsy-positive patients misclassified as negative. Among them, tuberculosis accounted for nine cases and malignancy for five cases. (2) Imprint cytology - False negatives (11 cases): These were biopsy-positive but imprint-negative. Among them, tuberculosis accounted for seven cases and malignancy for four cases.

This indicates that, while both cytology techniques missed some biopsy-positive cases, imprint cytology demonstrated fewer false negatives compared to brush cytology, especially in tuberculosis. Of the total, 19 patients without disease were falsely identified as positive (false positives), and 36 were correctly identified as negative (Table 2). In contrast, imprint cytology correctly identified 30 of the biopsy-positive cases and missed 11, showing fewer false negatives than brush cytology. Like brush cytology, imprint cytology had 19 false positives and 36 true negatives (Table 3). This comparison highlights that imprint cytology was able to capture a greater number of true disease cases while maintaining a comparable rate of false positives.

**Table 2:** Confusion Matrix - Brush Cytology vs. Histopathology

	Biopsy Positive (N = 41)	Biopsy Negative (N = 55)	Total
Test Positive	27 (65.85%)	19 (34.55%)	46
Test Negative	14 (34.15%)	36 (65.45%)	50
Total	41	55	96

**Table 3:** Confusion Matrix - Imprint Cytology vs. Histopathology

	Biopsy Positive (N = 41)	Biopsy Negative (N = 55)	Total
Test Positive	30 (73.17%)	19 (34.55%)	49
Test Negative	11 (26.83%)	36 (65.45%)	47
Total	41	55	96

To evaluate whether the observed difference between brush and imprint cytology was statistically significant, McNemar’s test was applied. The test statistic was 0.108 with a p-value of 0.742, indicating that the difference was not statistically significant. However, despite this lack of statistical difference, the higher sensitivity and NPV of imprint cytology suggest a clinically meaningful advantage over brush cytology.

Further diagnostic evaluation was performed using binary logistic regression to determine the predictive strength of each method. For brush cytology, a positive test result was associated with an odds ratio of 3.65 for having a positive biopsy result, indicating that such patients were 3.65 times more likely to have disease confirmed by histopathology. The model had a Pseudo R² value of 0.071, and the association was statistically significant, with a p-value of 0.0022. In comparison, imprint cytology yielded a stronger predictive model. A positive imprint cytology result corresponded to an odds ratio of approximately 5.17, suggesting a fivefold increased likelihood of histopathological confirmation. This model had a Pseudo R² of 0.110 and was highly statistically significant (p = 0.00014), indicating both superior explanatory power and stronger predictive validity than brush cytology (Table 4).

**Table 4:** Binary Logistic Regression Analysis of Cytological Techniques

Cytology Type	Odds Ratio	Pseudo R²	p-value	Log-Likelihood	Chi-Square (Test Statistic)	Interpretation
Brush Cytology	3.65	0.071	0.0022	-60.83	9.37	Significant predictive value
Imprint Cytology	5.17	0.110	0.00014	-58.29	14.45	Stronger predictive validity than the brush

**Figure 2:**
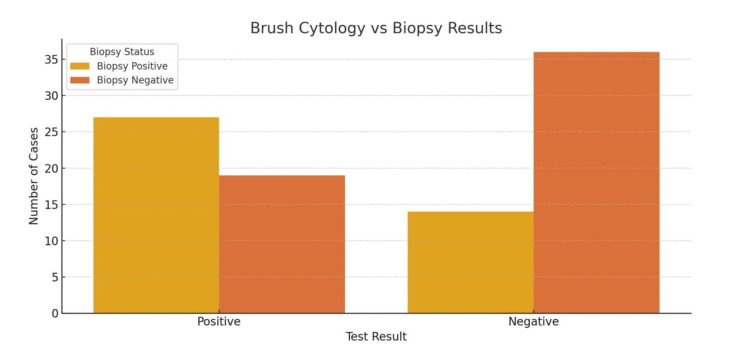
Confusion Matrix: Brush Cytology vs. Biopsy Results Data are represented as absolute numbers (N). Statistical significance was evaluated using McNemar’s test; p < 0.05 was considered significant.

**Figure 3:**
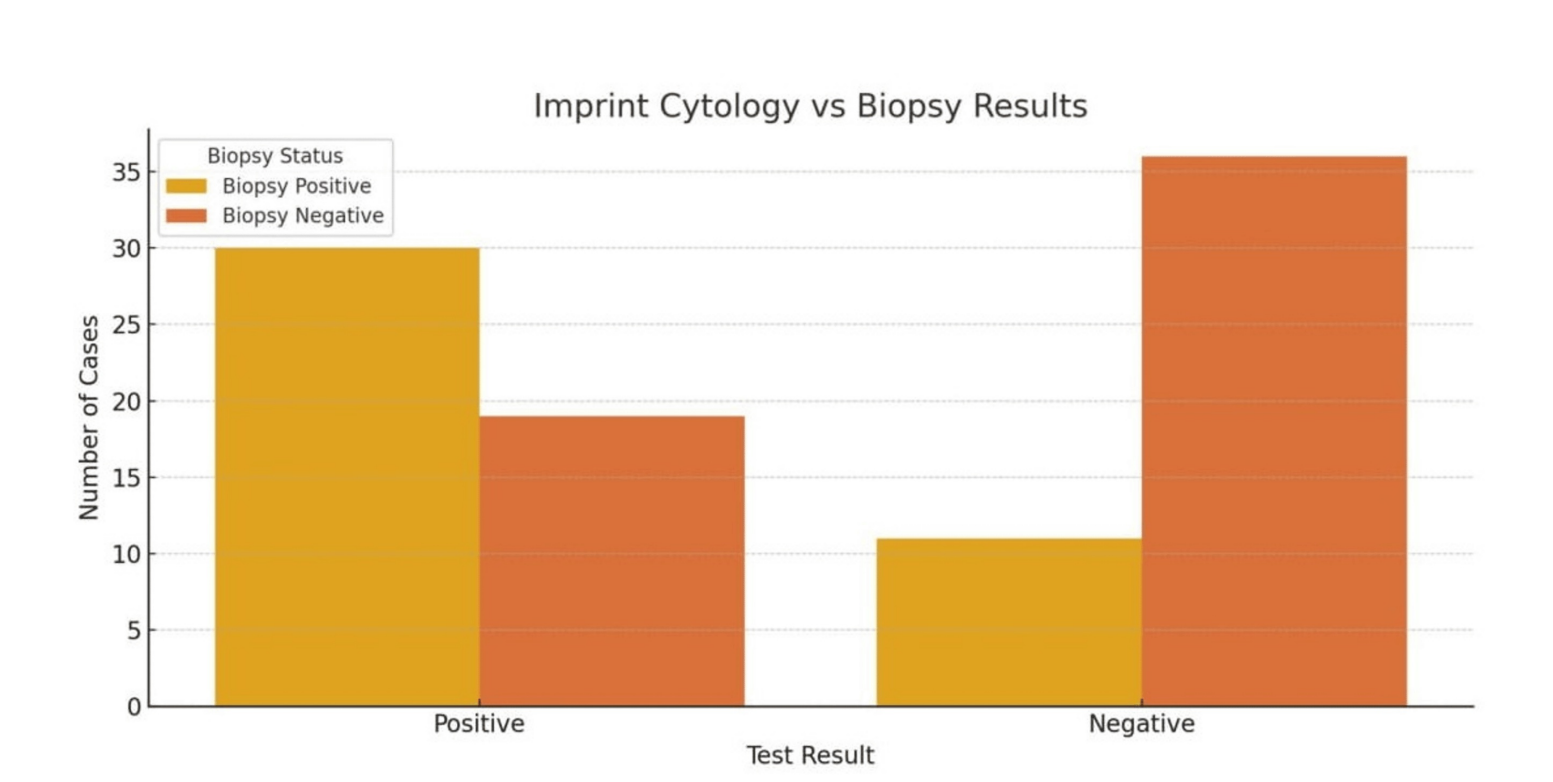
Confusion Matrix: Imprint Cytology vs. Biopsy Results Data are represented as absolute numbers (N). Statistical significance was evaluated using McNemar’s test; p < 0.05 was considered significant.

**Figure 4:**
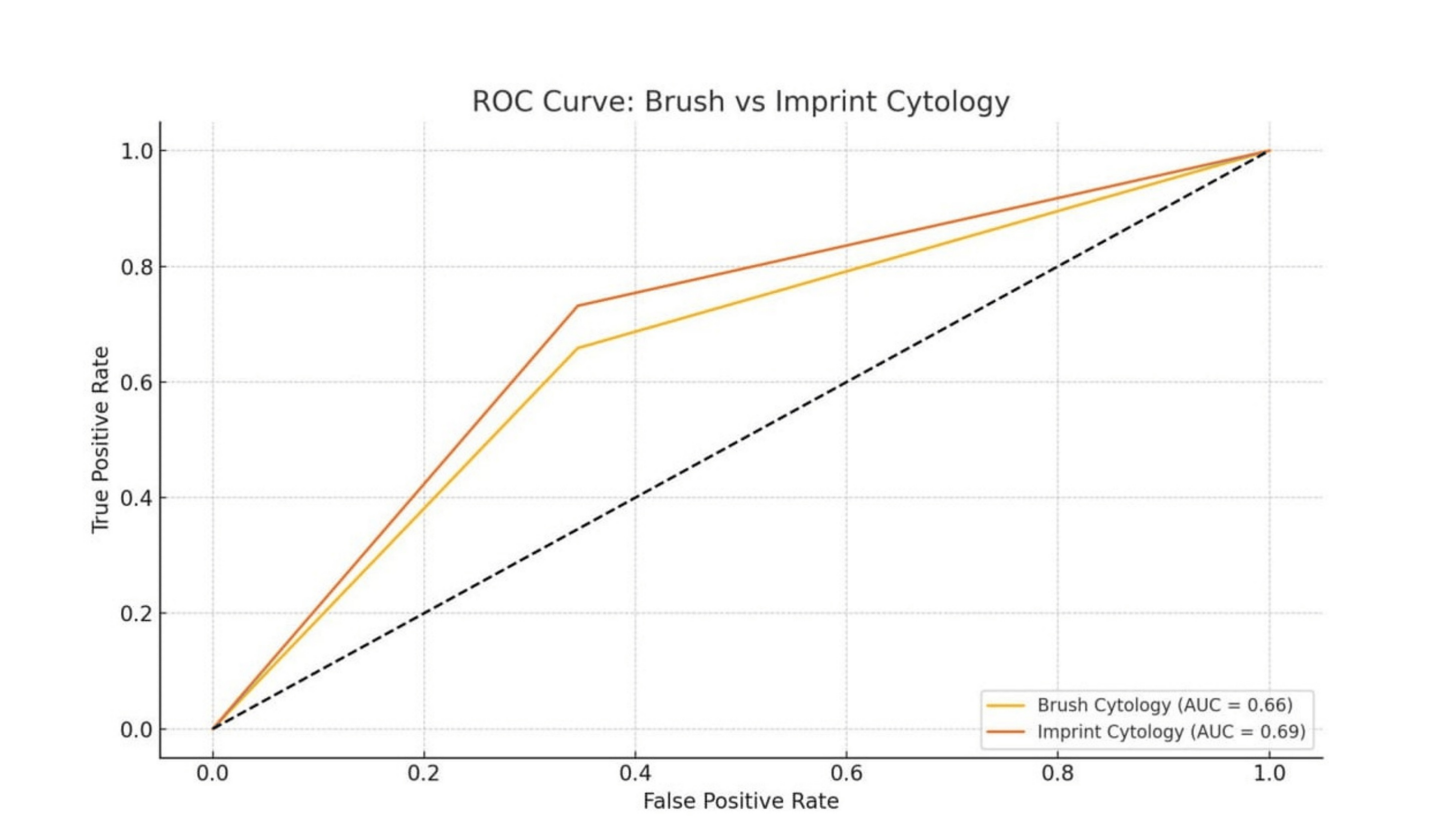
ROC Curve Comparison Between Brush and Imprint Cytology Data were analyzed using receiver operating characteristic (ROC) analysis, with performance assessed via area under the curve (AUC). Statistical significance was set at p < 0.05.

Additionally, ROC curve analysis was employed to assess the overall diagnostic performance of both techniques (Figures 2-3). Imprint cytology demonstrated a higher area under the curve (AUC) compared to brush cytology, further validating its greater accuracy across various diagnostic thresholds (Figure 4). This greater AUC reflects better sensitivity and specificity trade-offs for imprint cytology across all decision thresholds. This finding is consistent with other diagnostic metrics and strengthens the case for its clinical reliability in pleural biopsy evaluation.

In summary, while both brush and imprint cytology methods provided useful preliminary diagnostic information, imprint cytology showed a superior diagnostic profile. It not only identified more true positive cases but also exhibited better predictive strength and model fit in regression analysis. These findings support its potential role as a more effective adjunct to histopathological analysis in the diagnostic evaluation of pleural disease during thoracoscopy.

## Discussion

Our study highlights the diagnostic efficacy of imprint cytology compared to brush cytology in the evaluation of pleural biopsy specimens. With histopathology used as the gold standard, imprint cytology outperformed brush cytology in sensitivity, NPV, and predictive strength. Although McNemar’s test did not show statistical significance (p = 0.742), the trend in diagnostic yield favors imprint cytology.

The sensitivity of imprint cytology (73.17%) was higher than that of brush cytology (65.85%). Moreover, the NPV of imprint cytology (76.60%) implies that it is more reliable in ruling out disease. The logistic regression results further support the superiority of imprint cytology, with an odds ratio of approximately 5.17, compared to 3.65 for brush cytology. This indicates that a positive imprint result is over five times more likely to correlate with a positive biopsy result.

The higher Pseudo R² (0.110) and better model fit indices (lower AIC and higher log-likelihood) of the imprint model indicate its greater explanatory power in predicting biopsy results. ROC curve analysis also confirmed a larger AUC for imprint cytology, validating its superior diagnostic performance.

The ROC analysis also confirmed the diagnostic superiority of imprint cytology. The area under the ROC curve was greater for imprint cytology compared to brush cytology, reflecting improved test accuracy and reliability in predicting biopsy-confirmed disease. This means imprint cytology maintains better performance across different decision thresholds, making it more dependable for early diagnosis. These ROC findings, in conjunction with the regression and confusion matrix data, reinforce imprint cytology’s role as a valuable diagnostic adjunct during thoracoscopic procedures.

Our results are consistent with previous findings. Hantera et al. reported high sensitivity and diagnostic accuracy of imprint cytology in thoracoscopic pleural biopsies [1]. Similarly, Sharma et al. supported the use of cytological adjuncts for rapid diagnosis, particularly in malignancies [2]. These findings are particularly relevant in resource-limited settings, where immediate histopathological analysis may not be feasible.

Heine et al. and Metintas et al. further showed that imprint cytology during thoracoscopy had a diagnostic performance close to histopathology, and was superior to pleural fluid cytology, while brush cytology was found to be less sensitive [5,6].

A study by Prakash and Reiman demonstrated that cytologic techniques could outperform needle biopsy in malignant effusions, underscoring the importance of cytology as a complementary tool. In the thoracoscopic setting, imprint cytology offers a clear advantage over brush cytology, as it allows direct transfer of cells from freshly obtained pleural tissue, yielding intact clusters and better preservation of morphology [7].

Additionally, a study by Gupta et al. highlighted the excellent concordance between imprint cytology and histopathological diagnosis in thoracoscopic pleural biopsies, reinforcing the diagnostic value of imprint cytology in both malignant and granulomatous pleural conditions [8].

In a multicenter analysis by Wang et al., the integration of imprint cytology during thoracoscopic biopsies improved early diagnostic sensitivity for malignant pleural effusions from 64.5% to 81.3%, without adding significant cost or procedural time, supporting its real-world application [9]. Another study by Shaker et al. demonstrated a strong correlation between imprint cytology and histological subtyping in malignant pleural mesothelioma, which significantly improved early treatment stratification [10].

A major advantage of imprint cytology is its faster turnaround time. While histopathology may take several days, imprint cytology can provide preliminary results within hours, which is especially critical in malignancies where early therapeutic decisions are required. Its utility is further enhanced when interpreted in conjunction with gross thoracoscopic findings

To the best of our knowledge, this is the first study from India to evaluate imprint and brush cytology in pleural biopsy samples with such a large sample size (n = 96). Most previous Indian studies have included significantly smaller cohorts, limiting their generalizability.

Limitations

The present study has several limitations that must be acknowledged. Firstly, although the sample size of 96 patients is relatively large for a single-center study, broader multicentric validation would enhance the generalizability of the findings. Secondly, imprint cytology and brush cytology were interpreted by experienced personnel; therefore, diagnostic accuracy may vary depending on operator expertise and institutional resources. Finally, while the study demonstrated superior diagnostic performance of imprint cytology, it did not assess cost-effectiveness or turnaround time metrics in a quantifiable manner.

## Conclusions

Imprint cytology demonstrates superior diagnostic performance over brush cytology in pleural disease evaluation, using biopsy as the gold standard. Its rapid turnaround time, combined with high sensitivity and predictive power, makes it an ideal adjunctive diagnostic modality during thoracoscopic procedures. While histopathology remains the definitive diagnostic tool, imprint cytology offers a practical interim solution for early diagnosis and management - especially in high-volume or low-resource settings - for early confirmation of malignancy and tuberculosis, so that early removal of the intercostal drain and early discharge can be done. This study emphasizes that imprint cytology should be considered in each patient undergoing thoracoscopy, regardless of the presumed disease.
